# Data-sharing practices in publications funded by the Canadian Institutes of Health Research: implications for health sciences librarians

**DOI:** 10.29173/jchla29830

**Published:** 2025-12-01

**Authors:** David R. Scott, Sheilah C. Ayers, Kevin B. Read

**Affiliations:** 1 MLIS, MA, Health Sciences Librarian, University of Lethbridge, Lethbridge, AB; 2 MLIS; 3 MLIS, MAS, Associate Librarian, Research Data Management and Open Science, University of Saskatchewan, Saskatoon, SK

## Abstract

**Objective:**

Funding bodies such as Canada’s Tri-Agency have implemented requirements for grant recipients to encourage improved research data management (RDM) practices and data sharing. Consequently, RDM and data sharing have become a higher priority for researchers and stakeholders supporting the research process, including librarians. Health sciences research can present special challenges to those wishing to share and use research data, as access to sensitive data must be restricted. This study examines the data sharing practices of researchers funded by the Canadian Institutes of Health Research (CIHR) in recent years.

**Methods:**

We ran a search of PubMed Central to identify papers funded by CIHR that were published between 2020 and 2023 and had associated data. From the resulting records, we drew a sample of 368 articles. Using Qualtrics for each article, we recorded if and how data was shared and what types of documentation were provided alongside the data. Results were exported to and analyzed using Microsoft Excel.

**Results:**

We found that 69% of papers included a data availability statement. 34% of articles made at least some data readily accessible, while 31% indicated that some data was available via request or application. Only 9% of articles supplied the kinds of documentation that would support reuse of the data.

**Conclusion:**

Those seeking to reuse Canadian health sciences research data continue to face significant hurdles. We offer ideas for health sciences librarians looking to support researchers in their efforts to make data available and usable while respecting restrictions required due to ethical considerations.

## Introduction

In Canada, research data management (RDM), and consequently data sharing, have become increasingly important as they relate to research. In 2022, the Tri-Agency released its Research Data Management Policy [1], which requires researchers applying for agency funding to submit a data management plan (DMP), a living document that describes how research data is documented, formatted, stored, protected, preserved, and shared over the course of a research project. In the next phase of the policy’s implementation, the Tri-Agency aims to require that all research data, metadata, and code generated from research that has received Tri-Agency funding must be deposited in a digital repository [1].

To prepare for this requirement, several national initiatives have been undertaken to provide digital infrastructure for researchers who are looking to share their data. The Digital Research Alliance of Canada [2] launched the Federated Research Data Repository (FRDR) in 2019, a national repository service that accepts the deposit of and makes publicly available research data from Canadian researchers. FRDR provides a free platform for any Canadian researcher to submit data, should they need to comply with the upcoming data deposit implementation from the Tri-Agency. Similarly, in 2023 the University of Toronto, in partnership with the Digital Research Alliance of Canada, launched Borealis [3], a data repository network supported by academic libraries across Canada. It uses the Dataverse platform and is hosted by Scholars Portal and the University of Toronto Libraries [4]. Through Borealis, institutions host their own Dataverse, which allows their researchers to deposit research data locally, where it can subsequently be made discoverable in the national Borealis platform. These platforms ensure that Canadian researchers have a place to share their data publicly and that their research data is discoverable nationally.

While public data sharing has gained traction in research communities and yielded resources to support this traction in recent years, including in the health sciences, the deposit and sharing of sensitive data has garnered less attention. Sensitive data, which we define as data that may be used for research but whose access is limited due to ethical, commercial, or privacy concerns, is unable to be publicly shared. Accessing sensitive data typically requires an interested researcher to complete application forms, receive ethical, legal, or commercial approval, and sign disclosure and/or use agreements. In the health sciences, sensitive data—such as data collected from patients or human participants, populations, or health systems—is a common type of research data, with restrictions that limit its ability to be shared publicly, making it harder for researchers to access it as a result [5].

While data repositories like Borealis and the FRDR support finding and downloading publicly accessible research data, there are few platforms that exist to support the discovery and accessibility of sensitive data that could be acquired for future research [6]. In the health sciences, this often means that research data that is sensitive is undiscoverable. The challenges met when finding and accessing sensitive data in the health sciences are well documented: researchers cannot locate sensitive data because it is not indexed or available in an easily identifiable location; if a researcher does happen to find a sensitive dataset, the access request process is often not provided or unclear; the access request process is often time consuming; and finally, if a researcher does manage to navigate the access process and acquires a sensitive dataset, there are often poor RDM practices in place, such that there is not enough information or documentation to reuse the dataset [7–13]. These challenges have significant implications for health sciences research more broadly. For example, the inaccessibility of sensitive research data may result in research on human subjects being repeated unnecessarily, or researchers may limit their research questions because they know they cannot access a sensitive dataset that would yield more valuable results [14]. Barriers like those described above also impact meta-analyses by increasing the time burden for completion and limiting access to valuable data for analysis [15,16].

These concerns led two authors of this paper (DS, KR) to complete a study in 2021 examining if, how, and where researchers funded by the Canadian Institutes of Health Research (CIHR) share their data [7]. Examining data availability statements (DASs)— written descriptions authors include to indicate how to acquire their underlying research data—in CIHR-funded authors’ published articles, we found that of the 935 publications that stated data was available by application or request, not a single publication provided information about what the application or request process entailed. Further, when evaluating how well CIHR-funded authors applied RDM best practices to their shared datasets, we found that only 13.4% of more than 4000 articles provided documentation that would be required to understand and reuse that dataset. In the context of research data, documentation is defined as information that is created for the purposes of making a dataset understandable, reproducible, and reusable to another researcher. Examples of documentation can include, but are not limited to, metadata, readme files, data collection instruments, supplementary methods, and data analysis plans.

The results from this previous study, which highlight CIHR data sharing practices but also reflect the challenges researchers face in finding and accessing sensitive datasets, alongside the incoming Tri-Agency data deposit requirement, raise questions about how Canadian health sciences librarians can better support data sharing for their research communities. Health sciences librarians have been supporting RDM in many ways for more than a decade through training, consultation, and infrastructure [17–20]. RDM support in Canada has also grown exponentially during that time. Library support for researchers with respect to depositing and sharing sensitive data, however, is far less common. Our review of the literature was only able to find one key example, though provided in an American context, which is a health sciences library’s development of a data catalogue to improve the discovery of and access to sensitive data at an institutional level [21]. This effort demonstrates how health sciences libraries can support researchers by providing them with infrastructure that allows them to make their datasets discoverable using metadata, while keeping the data itself restricted and only available through a request and approval process.

Beyond libraries, large consortia [22] and individual researchers [23] have been seeking ways to improve sensitive data sharing more broadly through training, the development of ontologies, and stronger infrastructure. In Canada, the Digital Research Alliance has recently launched the Controlled Access Management project, which aims to collaborate with Canadian researchers to develop infrastructure and best practices to share sensitive data while controlling and managing access [24]. This new development presents an excellent opportunity for health sciences librarians to upskill and prepare to support researchers in depositing, managing, and providing clear access instructions for their sensitive data.

To gain more context about the data sharing behaviours of Canadian health sciences researchers, this paper provides a three-year update to the aforementioned project where we examined if, where, and how researchers funded by the CIHR share their data. This study highlights the locations where CIHR-funded data has been shared, identifies the current gaps in data sharing practices in a Canadian health sciences context, and discusses the implications for health sciences librarians concerning how our profession can support researchers who will be increasingly required to deposit and make discoverable their research data.

## Methods

### Data collection

**Fig. 1:**
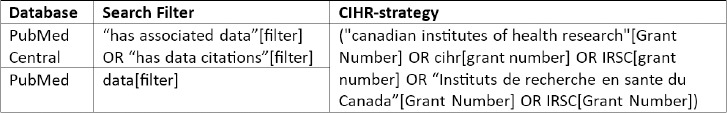
Search strategies

In many respects, our approach to this project replicated the methodology used in the previous study [5]. On July 19, 2023, we ran searches of PubMed and PubMed Central (PMC) to identify papers funded by CIHR that were published between January 1, 2020 and June 30, 2023, using filters to limit results to publications that indicated that they had associated data. (See Figure 1 for the search strings used in each database.)

We exported a total of 12 356 results (8356 from PMC and 4,000 from PubMed) to EndNote 20, where 1663 duplicates were removed using the Bramer method [25]. The remaining 10 693 records were then exported to an Excel file, where we assigned each a random number between one and one million and then sorted from smallest to largest. The top 371 articles were selected as our random sample (95% confidence interval).

**Fig. 2:**
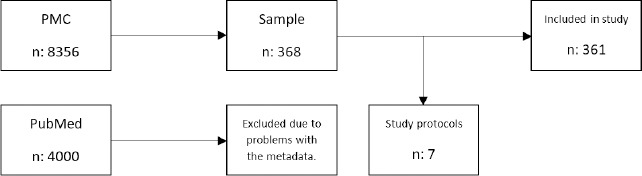
Flow diagram

After reviewing several dozen sample articles, we determined that due to errors in the PubMed metadata, we were limited to using the PMC records, exclusively. The errors in PubMed resulted from the “data” filter returning publications where the medical genetics information portal MedGen [26] was included as a data repository. MedGen is not a data repository, but rather an information portal for conditions, phenotypes, and findings related to medical genetics, and as a result skewed our PubMed sample. We also found that some PubMed records indicated data was available in a specific repository, but upon closer review, we could not find the data in the repository mentioned. Therefore, we used the 8,356 results retrieved from the July 19 PMC search, exported these to an Excel file, and using the same process described above drew a new sample of 368 articles (Figure 2).

We created a form using the August 2023 version of Qualtrics to record data gathered from the sample articles (Online Supplement, Appendix 1). For each article, we recorded whether the article was a protocol, whether a DAS was provided, if and how data was shared, and what types of documentation were provided alongside the data, if any.

Appendix 1

**Fig. 3:**
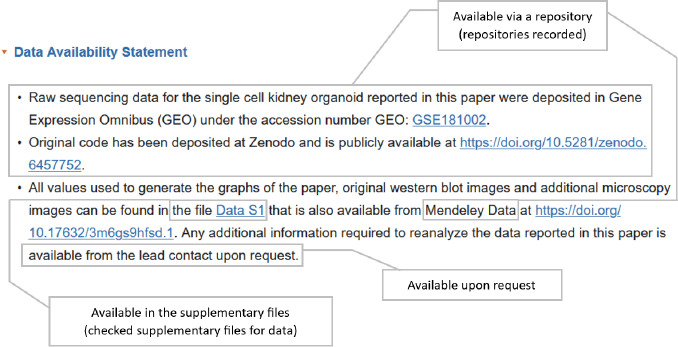
Example DAS

In recording data sharing, we examined the DAS and supplementary files and selected all applicable options for each article (Figure 3).

If the DAS specified that the data was available by request via an application process or that data sharing was not possible, we captured the reasons provided, if any. If data was made available through a website or repository, we recorded the name of the website or repository. We also noted instances when the DAS indicated that data was available in the supplementary files or main body of the article, but no data could be found there.

Finally, we recorded what documentation was either provided in the supplementary files or noted in the DAS. Documentation in our context was defined as any information that could be used to help interpret, understand, and/or use a dataset. Using data management best practice guidance [27], we noted if a publication used preservation formats for data that was shared (e.g., csv, xml, txt), or provided a data collection instrument, readme file, software code, data dictionary or codebook, study protocol, or DMP, as we determined that these types of documentation would best facilitate the reuse of shared data. We also noted other types of documentation including images, videos, supplementary figures and tables, or transparent reporting forms, because we felt they provide an additional level of understanding about the data, although they may not facilitate its reuse.

Supplementary figures and tables were considered documentation and not data because they represent summaries of the results within an article rather than the underlying data used to generate those results. Further, they are typically shared in formats that do not facilitate their reuse (e.g., PDF).

We exported the data from Qualtrics into an Excel spreadsheet, where, using the DOIs to match records, we added publication metadata (e.g., authors, article title, abstract) from EndNote to each record. After removing seven records because they were study protocols rather than research publications, we analyzed the remaining 361 results of the study using this spreadsheet (Online Supplements, Appendices 2 and 3).

Supplements, Appendices 2

Supplements, Appendices 3

### Data analysis

Two team members (DS and SA) reviewed the sample articles in PMC and extracted the data. To ensure consistency in how we interpreted and recorded information in the form, the reviewers analyzed the first 50 articles together. The remaining 318 were split evenly between the reviewers, who took note of papers for which any aspect of the data extraction was unclear. These situations were resolved through discussion involving the two reviewers and, in some instances, the third team member (KR).

Once the Qualtrics data and publication data from EndNote had been merged into a single Excel spreadsheet, we analyzed the results. Analysis involved simple counts and the calculation of proportions and ratios using Excel’s formula, find, sort, filter, and conditional formatting functions.

## Results

We examined the DASs and supplementary files of 361 articles supported by CIHR funding that were published between January 1, 2020 and June 30, 2023. Of these, 248 (69%) included a DAS. See Table 1 for a complete breakdown of data-sharing methods identified in this sample. Note that multiple data sharing methods may have been identified in each article.

**Table 1:** Publications by stated or identified data-sharing method (n=361 publications)

Data-sharing Method (not mutually exclusive)	Number (%) of Publications
Available upon request	46 (13)
Available upon reasonable request	50 (14)
Available via application	20 (6)
Available in the supplementary files^*^	81 (22)
Available in the contents of the published article^*^	51 (14)
Data sharing not applicable or possible	39 (11)
Data shared via a repository	86 (24)
Data shared via a website	19 (5)
No data was shared†	108 (30)

^*^ Includes articles with DASs indicating that data was available in the supplementary files and/or in the contents of the published article, but where we did not find any data †Excludes articles with DASs noting that data sharing was not applicable or possible

95 articles (26%) indicated that data was accessible through a repository or website. The repositories most frequently used were the Gene Expression Omnibus (GEO, 31), the Protein Data Bank (PDB, 20), and GitHub (18). See Table 2 for a list of all repositories used by at least two studies from the sample. Three studies used an institutional repository to share data.

**Table 2:** Data repositories used (n=95 publications)

Repository (not mutually exclusive)	Number (%) of Publications
Gene Expression Omnibus (GEO)	31 (33)
Protein Data Bank (PDB)	20 (21)
GitHub	18 (19)
ProteomeXchange	9 (9)
Figshare	5 (5)
Open Science Framework (OSF)	5 (5)
Sequence Read Archive (SRA)	5 (5)
BioProject	4 (4)
Electron Microscopy Data Bank (EMDB)	4 (4)
Genbank	4 (4)
Institutional repository	3 (3)
dbGaP	3 (3)
Mendeley Data	3 (3)
Zenodo	3 (3)
Biological Magnetic Resonance Data Bank (BMRB)	2 (2)
China Kadoorie Biobank	2 (2)
Ensembl	2 (2)
Genomic Data Commons	2 (2)

91 papers (25%) either made data accessible in the supplementary files or indicated in their DASs that data was available in the supplementary files or within the contents of the published article. Among those that indicated data was available in the supplementary files, we could find no data in the supplementary files in 19 instances. Seven articles indicated that data was available within the contents of the published article, but no data was found there. Eight articles did not have a DAS but did make some data accessible in the supplementary files. Altogether, 123 papers (34%) had at least some data readily accessible through a website, repository, or the supplementary files.

A total of 111 articles (31%) noted in their DASs that at least some data was available but not readily accessible. In most of these instances, data was noted to be available by request or reasonable request, typically to the corresponding author. Among the 20 publications that indicated data was available via application, 17 (n=17/20, 85%) provided reasons in the DAS for why an application was required. These reasons included confidentiality (n=8/17, 47%); the need to complete a data-access, data-transfer, or data-use agreement (n=4/17, 24%); general ethics concerns (n=3/17, 18%); license restrictions (n=3/17, 18%); repository regulations (n=1/17, 6%); and Indigenous data sovereignty rights (n=1/17, 6%). Three publications did not explain why an application was required.

Among the 39 publications (11%) indicating in their DASs that data sharing was not applicable or possible in at least some instances, 10 (n=10/39, 26%) cited confidentiality as the reason why data could not be shared. Two (n=2/39, 5%) explained that consent to share the data had not been obtained from participants. Two (n=2/39, 5%) pointed to proprietary restrictions as a barrier to data sharing. Three (n=3/39, 8%) noted that no data was collected for the publication. Four (n=3/39, 10%) provided other reasons, while one provided multiple reasons. 19 publications (n=19/39, 49%) provided no information concerning why data sharing was not possible or applicable.

Altogether, 161 studies (45%) neither made any data readily accessible nor indicated that any was available. This includes articles where we found no evidence or mention of data sharing (n=108), articles stating that data sharing was not applicable or possible in the DAS (n=27), and articles indicating in their DASs that the data was available within the supplementary files (n=19) or published article (n=7), for which we found no useable data in either location.

Documentation provided alongside articles most frequently included supplementary figures and tables (n=272 articles, 75%) and transparent reporting forms (n=43 articles, 12%). With respect to documentation that we identified as being suitable to support the reuse of data, we recorded no data management or analysis plans, and only two instances of software code and study protocols. (Researchers may have shared some forms of documentation through repositories and websites instead, such as software code through GitHub.) In total, 33 articles (9%) supplied the kinds of documentation that would support reuse of the data based on our criteria. See Table 3 for a full list of documentation recorded.

**Table 3:** Documentation and preservation formats identified (n=361 publications)

Type of Information	Number (%) of Publications
Supplementary figure/table	272 (75)
Transparent reporting form	43 (12)
Data collection instrument^*^	15 (4)
Image file	12 (3)
Video	10 (3)
Readme file^*^	9 (2)
Data dictionary/codebook^*^	4 (1)
Preservation format for structured data^*^	4 (1)
Study protocol^*^	2 (0)
Software code^*^	2 (0)
Data analysis plan/documentation^*^	0 (0)
Data management plan^*^	0 (0)

^*^ Documentation and preservation formats supporting the reuse of data

## Discussion

### Comparison of studies

In several respects, our results show no improvement in data sharing practices over the findings of the previous study [7]. In fact, a smaller proportion of studies in our sample shared their data publicly (34% versus 45%). A somewhat greater percentage indicated that data sharing was not applicable or possible (11% versus 7%). And relatively fewer articles included documentation in the supplementary files that would facilitate data reuse (9% versus 13%).

On the other hand, a greater proportion of sampled studies indicated that data was available via request or application (31% versus 23%). Additionally, of those articles noting in their DASs that data was available in the supplementary files, a much greater percentage actually did share data in this way (77% versus 28%).

Comparisons between the two studies should be made cautiously, as this study drew from and analyzed a random sample of eligible papers, and it drew articles only from PMC, whereas the previous study drew from both PMC and PubMed. But our results show that data sharing practices in Canadian health sciences research still have great strides to make. From the perspective of researchers wishing to access and use existing data, major hurdles remain. Often, when reviewing DASs that indicate data is available upon request, we found that it was difficult to determine what data is available and whether a researcher would be eligible to access it. As well, when data is shared, the documentation required to understand and use the data may not be provided. And when a DAS indicates that data is available upon request or by application, typically, few details are given about what that process would entail. These factors can severely limit researchers’ ability to find and use sensitive data for future projects.

### Data sharing and the health sciences librarian: implications for practice

Data sharing involves multiple stakeholders, and each can play a role in improving practice. As health sciences librarians, we can support the efforts of researchers in our subject areas to make data available and usable while respecting restrictions required due to ethical considerations. To skill up in the areas of RDM and data sharing, we encourage librarians to explore the Data Competencies established for health sciences librarians from the Medical Library Association and National Network of Libraries of Medicine [28]. These competencies were developed using a framework highlighting five key skills health sciences librarians can adopt to begin implementing data support services at their local institutions [29]. Librarians would also benefit from reviewing the chapter on sensitive data in the book *Research data management in a Canadian context*, which provides guidance on the types of sensitive data and their risk levels, as well as ethical considerations and policy guidance for researchers collecting or working with this data [30]. The Digital Research Alliance of Canada has also developed sensitive data toolkits [31–33] that can be used to inform researchers on how best to approach the management and sharing of these types of datasets.

As health sciences librarians in Canada, we can consider ways in which we might weave data sharing into our work. For instance, in addition to offering standalone workshops on the topic of RDM and data sharing, we can integrate data sharing guidance into our regular reference interactions and research consultations, information literacy instruction for graduate students, and library help guides designed for researchers [17,20,34,35], thereby leveraging existing relationships to promote data sharing platforms and practices [19]. As we develop and implement new ideas and programs, we can share what we’ve learned through conferences, journals, and other fora. Targeted training around sensitive data in health sciences research remains a particularly significant gap in the literature. There is little published in this area in Canada relative to the United States, but we have an opportunity to begin conversations around data sharing with our user communities, which may include asking questions such as have our researchers considered data sharing before? What challenges do they foresee in making their data available? If there are restrictions placed on sharing their data, what are they? How would they navigate a researcher request for their data? These questions can help us learn about our researchers’ unique contexts and identify ways to better support them.

Facilitating the discovery of and access to sensitive data within an institution often involves several procedural and governance processes requiring multiple stakeholders’ support. We recommend that health sciences librarians look to initiate conversations and develop partnerships with ethics boards, research administration, legal counsel, research computing, technology transfer groups, and affiliated partners at their local institutions around the topic of facilitating access to sensitive data. Health sciences librarians can serve as a core communication channel to share the challenges they hear from researchers and in turn disseminate institutional data sharing processes with their research community.

Canadian health sciences librarians can become familiar with and advocate for platforms such as FRDR and Borealis, if they have not already. If librarians have local installations of Borealis, they can advocate for the development of metadata records for sensitive research data stored on institutional storage systems to make this data more discoverable. Recent studies have explored the development of metadata schemas for sensitive datasets, but they have yet to be implemented [36]. Librarians can then begin discussions around improving data access workflows for sensitive data at an institutional level. To have success with sensitive data sharing, we want to reemphasize that the fostering of partnerships outside of the library—with IT, research offices, and disciplinary colleges, for example—to build support for data sharing infrastructure across our institutions is essential. A cohesive approach involving institutional stakeholders responsible for the ethical, legal, and security protections of research data is needed to ensure that formal institutional policies and guidelines are adhered to. Making sensitive data discoverable, providing transparent information about the access request process, and connecting that process to institutional infrastructure and workflows to offer secure access are ideal measures to successfully facilitate the sharing of sensitive data and require the coordinated efforts of these stakeholders across an institution. As mentioned in our introduction, this work has been undertaken at a U.S. institution where sensitive institutional data is discoverable and direct access request pathways are provided [21] but to our knowledge has yet to bear fruit in a Canadian context.

In collaborating with researchers navigating the sensitive data sharing landscape, we can offer guidance in writing effective DASs and DMPs. For example, we can encourage researchers to indicate in their DASs and DMPs what sensitive research data is available for secondary use and ensure that they provide access instructions and eligibility criteria indicating who can access this data and the required procedures for doing so. Systematic reviews, for example, have been found to have missing or inaccurate DASs [37]; we can ensure that any review we support or coauthor has a comprehensive and descriptive DAS. We can also go as far as encouraging researchers to share their DMPs alongside their published manuscripts to increase the transparency of their research data. A DMP template has already been integrated into the Digital Research Alliance’s DMP Assistant [38] that can be applied to systematic reviews. This additional information would fill the gap identified in prior research [37] and this paper’s finding that DMPs are not often included alongside the published manuscript.

Further, we can demonstrate data sharing best practices through our own research, including projects we initiate and lead in the field of health sciences librarianship. We can share data where applicable and appropriate, as well as develop and share documentation to facilitate the reuse of data. Journals that support health sciences librarianship have adopted data sharing policies to encourage sharing in this way [39,40]. Developing strong data documentation like readme files, codebooks, and analysis plans to improve the understandability of research data allows our research projects to serve as examples for the research communities we support. While our projects may not involve the sensitive data with which many health sciences researchers are working, gaining first-hand experience developing a DMP, building data documentation, and sharing our own data can provide us with insight into the process and some of the challenges researchers encounter when working to make their data findable, accessible, and usable.

### Limitations

We acknowledge that the differences noted in our comparison of the results from our previous work may in part may be impacted by the random sampling approach we chose in this study. As we did not attempt to locate data in repositories or other websites (unlike the supplementary files), we cannot confirm that data was available from these sources when authors indicated so. Similarly, if additional documentation was provided alongside the data on these platforms, it was not captured in our study.

### Conclusion

The responsibility to share health sciences research data ethically, including sensitive data, must ultimately fall to the researchers. As librarians, however, we can encourage good data sharing practices, liaise with key stakeholders at our institutions to provide researchers with clarity on the steps they must take to share or provide access to sensitive data, and ensure that many of the necessary supports are in place for those researchers who would like to have their data available and discoverable for others to use.

As the Tri-Agency continues to advance its RDM policy and implements the requirement that all funded research data be deposited in a digital repository, updates to this study would be informative. Of particular significance to health sciences librarians, research concerning RDM practices in knowledge synthesis projects would likewise be welcome. We also believe a closer examination of how journals guide, review, and enforce the content provided in DASs, especially when sensitive data has been collected, would shed light on how and why researchers choose to include certain information in their DASs.

## Data Availability

The Qualtrics form used to extract data for this study may be found in Appendix 1 in the online supplementary files. The data extracted from the Qualtrics form and EndNote, which was analyzed to produce this study’s results, may be found in an Excel spreadsheet and CSV file in Appendices 2 and 3 in the online supplementary files. The corresponding author welcomes inquiries concerning these files or any other aspect of the study.

## References

[1] Government of Canada. Tri-Agency research data management policy [Internet]. Ottawa: Government of Canada; 2021 Mar 14 [cited 2025 Aug 22]. Available from: http://science.gc.ca/eic/site/063.nsf/eng/h_97610.html.

[2] Digital Research Alliance of Canada. Canadian Digital Research Infrastructure Investment Overview 2023-25 [Internet]. Toronto: Digital Research Alliance of Canada; 2022. Available from: https://web.archive.org/web/20250422010550/ https://www.alliancecan.ca/sites/default/files/2023-03/2023-25%20DRI%20Investment%20Overview.pdf.

[3] Borealis. Borealis: the Canadian dataverse repository [Internet]. [place unknown]: Borealis; [cited 2025 Jun 16]. Available from: https://borealisdata.ca/.

[4] Scholars Portal [Internet]. [Toronto]: Ontario Council of University Libraries; [date unknown] [cited 2025 Jun 16]. Available from: https://scholarsportal.info/.

[5] Canadian Institutes of Health Research. CIHR Health Research and Health-Related Data Framework and Action Plan [Internet]. [Ottawa]: Government of Canada; 2017 [cited 2025 Jun 16]. Available from: https://cihr-irsc.gc.ca/e/50182.html.

[6] Cobey K, Roche D, Ripp C, Dhane F, Armond A, Wang M, et al. Cross-sectional analysis of Canadian institutional RDM strategies. [cited 2025 Jun 16]. In: Open Science Framework [Internet]. [place unknown]: OSF. 2023 Nov 8; Available from: https://osf.io/fpgqn/.

[7] Read KB, Ganshorn H, Rutley S, Scott DR. Data-sharing practices in publications funded by the Canadian Institutes of Health Research: a descriptive analysis. Can Med Assoc Open Access J [Internet]. 2021 Nov 9;9(4):E980–7. Available from: 10.9778/cmajo.20200303. PMC8580829 34753787

[8] Read KB, Gibson G, Leahey A, Peterson L, Rutley S, Shi J, et al. Understanding the challenges associated with finding and accessing restricted data in Canada: a mixed methods study. FACETS [Internet]. 2024 Feb 22;9:1–9. Available from: 10.1139/facets-2023-0102.

[9] Gabelica M, Bojčić R, Puljak L. Many researchers were not compliant with their published data sharing statement: mixed-methods study. J Clin Epidemiol [Internet]. 2022 Oct;150:35-41. Available from: 10.1016/j.jclinepi.2022.05.019. 35654271

[10] Lugg-Widger FV, Angel L, Cannings-John R, Hood K, Hughes K, Moody G, et al. Challenges in accessing routinely collected data from multiple providers in the UK for primary studies: managing the morass. Int J Popul Data Sci [Internet]. 2018 Sep 12;3(3):432. Available from: 10.23889/ijpds.v3i3.432. 34095522 PMC8142952

[11] Bonomi L, Huang Y, Ohno-Machado L. Privacy challenges and research opportunities for genomic data sharing. Nat Genet [Internet]. 2020 Jun 29;52(7):646–54. Available from: 10.1038/s41588-020-0651-0. 32601475 PMC7761157

[12] Prince K, Jones M, Blackwell A, Simpson A, Meakins S, Vuylsteke A. Barriers to the secondary use of data in critical care. J Intensive Care Soc [Internet]. 2018;19(2):127–31. Available from: 10.1177/1751143717741082. 29796069 PMC5956691

[13] Byrd JB, Greene AC, Prasad DV, Jiang X, Greene CS. Responsible, practical genomic data sharing that accelerates research. Nat Rev Genet [Internet]. 2020 Jul 21;21(10):615–29. Available from: 10.1038/s41576-020-0257-5. 32694666 PMC7974070

[14] van Schaik TA, Kovalevskaya NV, Protopapas E, Wahid H, Nielsen FG. The need to redefine genomic data sharing: a focus on data accessibility. Appl Transl Genomics [Internet]. 2014 Sep 28;3(4):100–4. Available from: 10.1016/j.atg.2014.09.013. PMC4888834 27294022

[15] Polanin JR, Williams RT. Overcoming obstacles in obtaining individual participant data for meta-analysis. Res Synth Methods [Internet]. 2016 Sep 7;7(3):333–41. Available from: 10.1002/jrsm.1208. 27228953

[16] Rogozińska E, Marlin N, Thangaratinam S, Khan KS, Zamora J. Meta-analysis using individual participant data from randomised trials: opportunities and limitations created by access to raw data. Evid Based Med [Internet]. 2017 Oct;22(5):157–62. Available from: 10.1136/ebmed-2017-110775. 28818966

[17] Kim S, Syn SY. Practical considerations for a library’s research data management services: the case of the National Institutes of Health Library. J Med Libr Assoc [Internet]. 2021 Jul 1;109(3):450-8. Available from: 10.5195/jmla.2021.995. 34629974 PMC8485941

[18] Silkotch C, Garcia-Milian R, Hersey D. Partnering with health sciences libraries to address challenges in bioimaging data management and sharing. Histochem Cell Biol [Internet]. 2023 Sep;160(3):193–8. Available from: 10.1007/s00418-023-02198-1. 37247072

[19] Goldman J, Muilenburg J, Schorr AN, Ossom-Williamson P, Uribe-Lacy CJ. Trends in research data management and academic health sciences libraries. Med Ref Serv Q [Internet]. 2023 Jul 3;42(3):273–93. Available from: 10.1080/02763869.2023.2218776. 37459491 PMC10405786

[20] Read KB, Koos J, Miller RS, Miller CF, Phillips GA, Scheinfeld L, et al. A model for initiating research data management services at academic libraries. J Med Libr Assoc [Internet]. 2019 Jul 1;107(3):432-41. Available from: 10.5195/jmla.2019.545. 31258450 PMC6579580

[21] Yee M, Surkis A, Lamb I, Contaxis N. The NYU Data Catalog: a modular, flexible infrastructure for data discovery. J Am Med Inform Assoc [Internet]. 2023 Oct;30(10):1693-700. Available from: 10.1093/jamia/ocad125. 37414539 PMC10531119

[22] Jang JB, Pienta AM, Levenstein MC, Saul J. Restricted data management: The current practice and the future. J Priv Confidentiality [Internet]. 2023 Dec 6;13(2). Avilable from: 10.29012/jpc.844. PMC10956935 38515607

[23] Martorana M, Kuhn T, Siebes R, Van Ossenbruggen J. Advancing data sharing and reusability for restricted access data on the Web: introducing the DataSet-Variable Ontology. In: Proceedings of the 12th Knowledge Capture Conference 2023 [Internet]. Pensacola (FL): ACM; 2023 [cited 2025 Jun 16]. p. 83–91. Available from: https://dl.acm.org/doi/10.1145/3587259.3627559.

[24] Digital Research Alliance of Canada. Controlled access management for research data [Internet]. Toronto: Digital Research Alliance of Canada; [cited 2025 Jun 16]. Available from: https://web.archive.org/web/20250315182531/ https://alliancecan.ca/en/funding-opportunities/controlled-access-management-research-data.

[25] Bramer WM, Giustini D, De Jonge GB, Holland L, Bekhuis T. De-duplication of database search results for systematic reviews in EndNote. J Med Libr Assoc [Internet]. 2016 Jul;104(3):240-3. Available from: 10.3163/1536-5050.104.3.014. 27366130 PMC4915647

[26] MedGen [Internet]. Bethesda (MD): National Library of Medicine (US). [date unknown] - [cited 2025 Jun 16]. Available from: https://www.ncbi.nlm.nih.gov/medgen?Db=medgen.

[27] Thompson K, Hill E, Carlisle-Johnston E, Dennie D, Fortin É. Research data management in the Canadian context [Internet]. London (ON): Western Libraries, Western University; 2023 [cited 2024 Apr 5]. Available from: https://ecampusontario.pressbooks.pub/canadardm/.

[28] NNLM. Data competencies [Internet]. Bethesda (MD): National Library of Medicine (US); [updated 2025 Jul 21; cited 2025 Jun 16]. Available from: https://www.nnlm.gov/guides/data-competencies.

[29] Federer L, Foster ED, Glusker A, Henderson M, Read K, Zhao S. The Medical Library Association Data Services Competency: a framework for data science and open science skills development. J Med Libr Assoc [Internet]. 2020 Apr;108(2):304-9. Available: 10.5195/jmla.2020.909. 32256242 PMC7069817

[30] Rod DAB, Thompson K. Sensitive data: practical and theoretical considerations. In: Thompson K Hill E Carlisle-Johnston E Dennie D Fortin É. Research data management in the Canadian context [Internet]. London (ON): Western Libraries, Western University; 2023 [cited 2025 Jun 16]. [about 28 p.]. Available from: https://ecampusontario.pressbooks.pub/canadardm/chapter/sensitive-data-practical-and-theoretical-considerations/.

[31] Sensitive Data Expert Group. Sensitive data toolkit for researchers part 1: glossary of terms for sensitive data used for research purposes. [cited 2025 Jun 19]. In: Zenodo [Internet]. [place unknown]: CERN. 2020 Sep 30; Available from: https://zenodo.org/records/4088946.

[32] Sensitive Data Expert Group. Sensitive data toolkit for researchers part 2: human participant research data risk matrix. [cited 2025 Jun 19]. In: Zenodo [Internet]. [place unknown]: CERN. 2020 Oct 1; Available from: https://zenodo.org/records/4088954.

[33] Sensitive Data Expert Group. Sensitive data toolkit for researchers part 3: research data management language for informed consent. [cited 2025 Jun 19]. In: Zenodo [Internet]. [place unknown]: CERN. 2020 Oct 1; Available from: https://zenodo.org/records/4107178.

[34] Tenopir C, Sandusky RJ, Allard S, Birch B. Research data management services in academic research libraries and perceptions of librarians. Libr Inf Sci Res [Internet]. 2014 Apr;36(2):84–90. Available from: 10.1016/j.lisr.2013.11.003.

[35] Sheikh A, Malik A, Adnan R. Evolution of research data management in academic libraries: A review of the literature. Inf Dev [Internet]. 2025 Jun;41(2):305–19. Available from: 10.1177/02666669231157405.

[36] Read KB, Gibson G, Leahey A, Peterson L, Rutley S, Shi J, et al. Identifying metadata commonalities across restricted health data sources: A mixed methods study exploring how to improve the discovery of and access to restricted datasets. J EScience Librariansh [Internet]. 2024 Aug 16;13(2). Available from: 10.7191/jeslib.907.

[37] Page MJ, Nguyen PY, Hamilton DG, Haddaway NR, Kanukula R, Moher D, et al. Data and code availability statements in systematic reviews of interventions were often missing or inaccurate: a content analysis. J Clin Epidemiol [Internet]. 2022 Jul;147:1–10. Available from: 10.1016/j.jclinepi.2022.03.003. 35278609

[38] Ganshorn H, Premji Z, Ronksley PE. Data management plan template: systematic reviews. [cited 2025 Jun 16]. In: Zenodo [Internet]. [place unknown]: CERN. 2023 Mar 30; Available from: https://zenodo.org/records/7786940.

[39] Read K, Campbell A, Kitchin V, MacDonald H, McKeown S. Embracing the value of research data: introducing the JCHLA/JABSC Data Sharing Policy. J Can Health Libr Assoc [Internet]. 2021 Apr 2;42(1):6-13. Available from: 10.29173/jchla29536. 35949502 PMC9327608

[40] Akers KG, Read KB, Amos L, Federer LM, Logan A, Plutchak TS. Announcing the Journal of the Medical Library Association’s data sharing policy. J Med Libr Assoc [Internet]. 2019 Oct 1;107(4):468-71. Available from: 10.5195/jmla.2019.801. 31607804 PMC6774558

