# Supplementary material for: Data-sharing practices in publications funded by the Canadian Institutes of Health Research: implications for health sciences librarians
Source: J Can Health Libr Assoc. 2025 Dec 1;46(3):104–14. doi: 10.29173/jchla29830 (PMC13157968; doi:10.29173/jchla29830)
Supplement: Supplementary file 1 [file JCHLA-46-104-s001.pdf]

Appendix 1: Data Collection Instrument (Qualtrics form)

CIHR RDM Update Research Project

Start of Block: Default Question Block

Q1 DOI (Copy from Excel spreadsheet.)

____

Q2 Collaborator Responsible

▢ Author name (1)

▢ Author name (2)

Q3 Institutional Affiliation

- ☐ Acadia University (1)
- ☐ University of Alberta (2)
- ☐ Algoma University (3)
- ☐ Athabasca University (4)
- ☐ Bishop's University (5)
- ☐ Brandon University (6)
- ☐ University of British Columbia (7)
- ☐ Brock University (8)
- ☐ University of Calgary (9)
- ☐ Cape Breton University (10)
- ☐ Capilano University (11)
- ☐ Carleton University (12)
- ☐ Concordia University (13)
- ☐ Dalhousie University (14)
- ☐ Dominican University College (15)
- ☐ École de technologie supérieure (16)
- ☐ École nationale d'administration publique (17)

- [ ] Emily Carr University of Art and Design (18)
- [ ] First Nations University of Canada (19)
- [ ] University of the Fraser Valley (20)
- [ ] University of Guelph (21)
- [ ] HEC Montréal (22)
- [ ] Huron University College (23)
- [ ] Institut national de la recherche scientifique (24)
- [ ] University of King's College (25)
- [ ] Kwantlen Polytechnic University (26)
- [ ] Lakehead University (27)
- [ ] Laurentian University (28)
- [ ] University of Lethbridge (29)
- [ ] MacEwan University (30)
- [ ] University of Manitoba (31)
- [ ] McGill University (32)
- [ ] McMaster University (33)
- [ ] Memorial University of Newfoundland (34)
- [ ] Mount Alison University (35)

- [ ] Mount Royal University (36)
- [ ] Mount Saint Vincent University (37)
- [ ] Nipissing University (38)
- [ ] University of Northern British Columbia (39)
- [ ] Nova Scotia College of Art and Design University (40)
- [ ] Ontario College of Art and Design University (41)
- [ ] University of Ottawa (42)
- [ ] Polytechnique Montréal (43)
- [ ] Queen's University (44)
- [ ] Royal Military College of Canada (45)
- [ ] Royal Roads University (46)
- [ ] Saint Francis Xavier University (47)
- [ ] Saint Mary's University (48)
- [ ] Saint Paul University (49)
- [ ] Université Sainte-Anne (50)
- [ ] Simon Fraser University (51)
- [ ] St. Thomas University (52)
- [ ] Thompson Rivers University (53)

- [ ] University of Toronto (54)
- [ ] Toronto Metropolitan University (55)
- [ ] Trent University (56)
- [ ] Université de Moncton (57)
- [ ] Université de Montréal (58)
- [ ] Université de Sherbrooke (59)
- [ ] Université du Québec à Chicoutimi (60)
- [ ] Université du Québec à Montréal (61)
- [ ] Université du Québec à Rimouski (62)
- [ ] Université du Québec à Trois-Rivières (63)
- [ ] Université du Québec en Abitibi-Témiscamingue (64)
- [ ] Université du Québec en Outaouais (65)
- [ ] Université Laval (66)
- [ ] University College of the North (67)
- [ ] University of New Brunswick (68)
- [ ] University of Prince Edward Island (69)
- [ ] University of Regina (70)
- [ ] University of Saskatchewan (71)

- ☐ Ontario Tech University (72)
- ☐ Vancouver Island University (73)
- ☐ University of Victoria (74)
- ☐ University of Waterloo (75)
- ☐ Western University (76)
- ☐ Wilfrid Laurier University (77)
- ☐ University of Windsor (78)
- ☐ University of Winnipeg (79)
- ☐ Université de Saint-Boniface (80)
- ☐ York University (81)
- ☐ Other (specify) (82) ____

Q4 Is this article a study protocol?

- ○ Yes (1)
- ○ No (2)

Q5 Does the article include a data availability statement?

- ○ Yes (1)
- ○ No (2)

Q6 How was the data shared?

- ☐ Available upon request [DAS only] (1)
- ☐ Available upon reasonable request [DAS only] (2)
- ☐ Available upon request via application [DAS only] (3)
- ☐ Available via a repository (4)
- ☐ Available via a website (5)
- ☐ Available in the supplementary files (6)
- ☐ All data is available within the contents of the published article [DAS only] (7)
- ☐ Data sharing is not applicable/possible [DAS only] (8)
- ☐ Data will be made available at a future date [DAS only] (9)
- ☐ No data was shared (10)
- ☐ Other (specify) (11) ____

Display This Question:

If How was the data shared? = Available upon request via application [DAS only]

Q7 What reasons were provided in the data availability statement for why an application was required to access the data?

- ▢ Confidentiality (1)
- ▢ Data access/transfer/use agreement (2)
- ▢ Ethics (3)
- ▢ Indigenous considerations (4)
- ▢ License restrictions (5)
- ▢ No details provided (6)
- ▢ Other (specify) (7) ____

Display This Question:

If How was the data shared? = Available via a repository

Q8 What is the name of the repository/repositories provided in the data availability statement where data was shared?

- ☐ BioProject (1)
- ☐ Dataverse (2)
- ☐ dbGaP (3)
- ☐ dbSNP (4)
- ☐ dbVar (5)
- ☐ Dryad (6)
- ☐ European Genome-Phenome Archive (7)
- ☐ figshare (8)
- ☐ Flybase (9)
- ☐ FRDR (10)
- ☐ GDB (11)
- ☐ Genbank (12)
- ☐ Genomic Data Commons (13)
- ☐ GEO (14)
- ☐ GitHub (15)
- ☐ ICPSR (16)
- ☐ Institutional Repository (17)

- [ ] ISRCTN (18)
- [ ] Mendeley Data (19)
- [ ] Neuromorpho (20)
- [ ] Neuroscience Information Framework (NIF) (21)
- [ ] NIMH Data Archive (22)
- [ ] OMIM (23)
- [ ] Open Science Framework (24)
- [ ] PDB (25)
- [ ] PIR (26)
- [ ] PubChem-Bioassay (27)
- [ ] PubChem-Compound (28)
- [ ] PubChem-Substance (29)
- [ ] RefSeq (30)
- [ ] SRA (31)
- [ ] SWISSPROT (32)
- [ ] The Cancer Imaging Archive (33)
- [ ] UniMES (34)
- [ ] UniParc (35)

☐ UniProtKB (36)

☐ UniRef (37)

☐ Vivli (38)

☐ Wormbase (39)

☐ Zebrafish Model Organism Database (ZFIN) (40)

☐ Zenodo (41)

☐ Other (specify) (42) ____

Display This Question:

If How was the data shared? = Available via a website

Q9 What is the name of the website(s) provided in the data availability statement where data was shared?

____

Display This Question:

If How was the data shared? = Data sharing is not applicable/possible [DAS only]

Q10 What reasons were provided in the data availability statement for not sharing the data?

- ☐ Confidentiality (1)
- ☐ No consent obtained (2)
- ☐ Ethical reasons other than consent (3)
- ☐ Indigenous considerations (4)
- ☐ No additional information was provided (5)
- ☐ No data was collected within the publication (6)
- ☐ Proprietary restrictions (7)
- ☐ Other (specify) (8) ____

Q11 Please select any data-related information provided in the article (e.g., associated data section, acknowledgements, supplementary files) or noted in the DAS:

- ▢ Data analysis plan/documentation (only when indicated as one) (1)
- ▢ Data collections instruments (e.g., survey, interview questions, data extraction file) (2)
- ▢ Data dictionary/codebook (3)
- ▢ Data files (e.g., raw or processed quantitative or qualitative data) (4)
- ▢ Data management plan (only when indicated as one) (5)
- ▢ Readme files (6)
- ▢ Image files (7)
- ▢ Preservation formats for structured data (e.g., csv, tab-delimited, xml, txt) (8)
- ▢ Software code (9)
- ▢ Study protocol (10)
- ▢ Supplementary figures and/or tables (11)
- ▢ Transparent reporting form (includes PRISMA) (12)
- ▢ Videos (13)
- ▢ Other (specify) (14) ____

Q13 Please insert any additional comments that were not covered by the questions above.

____

End of Block: Default Question Block
